# Coronavirus Disease 2019 (COVID-19)-Associated Thromboembolic Disease: A Report of Three Patients With Pulmonary Embolism

**DOI:** 10.7759/cureus.8583

**Published:** 2020-06-12

**Authors:** Jeffrey Guccione, Daniel Ocazionez, Gabriel Aisenberg, Erika Odisio

**Affiliations:** 1 Diagnostic Radiology, University of Texas Health Science Center at Houston, Houston, USA; 2 Internal Medicine, John P. and Kathrine G. McGovern Medical School, University of Texas Health Science Center at Houston, Houston, USA

**Keywords:** pulmonary embolism (pe), acute pulmonary embolism, sars-cov-2, coagulopathy, covid coagulopathy, covid-19

## Abstract

Coagulopathy and thromboembolic disease, including pulmonary embolism (PE), are reported complications of coronavirus disease 2019 (COVID-19). The mechanism is not fully understood. We present three patients with COVID-19 and concurrent PE.

## Introduction

The coronavirus disease 2019 (COVID-19) pandemic has entered the sixth month of its course since it was first reported locally in Wuhan, China on December 31, 2019. Among the varied manifestations of this disease, thromboembolic complications, including pulmonary embolism (PE), have been reported. A recent study of 100 patients with severe acute respiratory syndrome coronavirus 2 (SARS-CoV-2), who received a CT angiogram PE (CTA-PE) protocol, found that 23% had PE [1]. There is significant overlap in the typical symptoms and laboratory data among patients with COVID-19, those with PE, and individuals with hypoxic respiratory failure in the context of sepsis. We report three cases of patients with COVID-19 complicated by PE to help illustrate this connection.

## Case presentation

Patient 1

**Figure 1:**
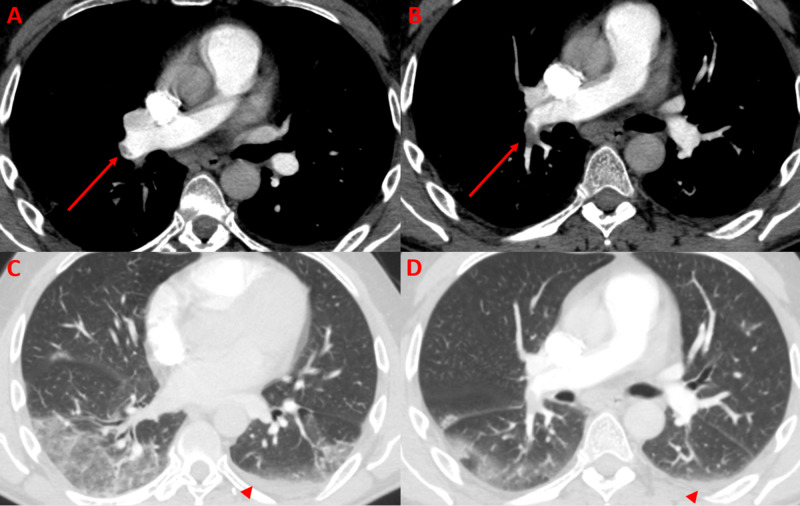
Axial CTA-PE protocol of the chest (A, B) shows an occlusive thrombus in the proximal right lower lobar pulmonary artery (arrows). (C, D) Lung windows demonstrate peripheral ground-glass opacities in the dependent lungs with a basilar and right lung predominance. A small left-sided pleural effusion is also present (arrowheads). CTA-PE, CT angiogram pulmonary embolism

A 59-year-old male airline worker with no past medical history presented with 10 days of fever, cough, shortness of breath, and vomiting. He had recently traveled to Europe and Asia and had contact with a confirmed COVID-19 patient. He was admitted and tested for SARS-COV-2, which returned negative; the test was repeated on hospital day 5 due to a high index of suspicion. While awaiting the test results, he developed new chest tightness and worsening shortness of breath on day 6, prompting a CTA-PE protocol. This demonstrated right lower lobar and segmental pulmonary emboli with dependent subpleural ground-glass opacities in the distribution of the emboli (Figure 1).

A venous Doppler ultrasound (US) of the lower extremities was negative for deep vein thrombosis (DVT). Heparin, azithromycin, and hydroxychloroquine treatment was initiated. He developed hypoxic respiratory failure requiring intubation on hospital day 10. Soon after, his repeat test returned positive for SARS-CoV-2. On day 11, he developed pneumomediastinum with extensive subcutaneous emphysema and small bilateral pneumothoraces. Due to decompensated respiratory failure, venovenous extracorporeal membrane oxygenation (ECMO) was initiated. Immediately thereafter, the mechanical oxygenator thrombosed, prompting emergent exchange and supportive care. A D-dimer level was obtained on admission to the ICU and found to be elevated to 1.34 mg/ml, eventually peaking at >20 mg/ml. He has remained on ECMO with an inferior vena cava (IVC) thrombus identified on US during catheter exchange on hospital day 54, despite anticoagulation. He subsequently underwent mechanical retrieval of the thrombus along with placement of an IVC filter.

Patient 2

**Figure 2:**
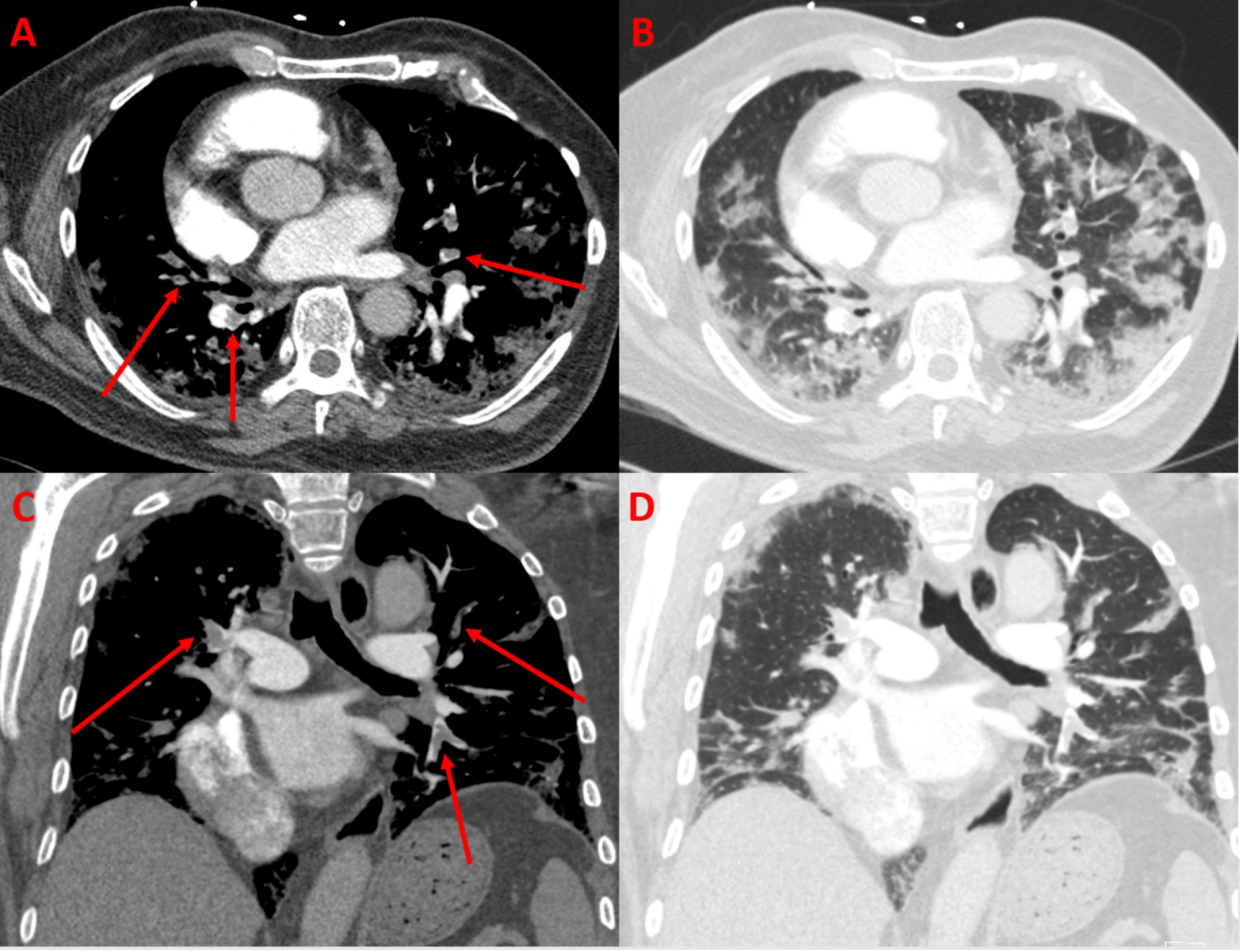
CTA-PE protocol of the chest with axial (A, B) and coronal (C, D) views show numerous filling defects in the bilateral lobar and segmental pulmonary arteries (arrows) consistent with pulmonary emboli. Lung windows show peripheral predominant ground-glass and consolidative opacities (B, D). CTA-PE, CT angiogram pulmonary embolism

A 68-year-old male with hypertension, dextrocardia, and obesity presented two weeks after a positive outpatient SARS-CoV-2 test with shortness of breath, cough, and loss of taste. He had been isolating at home with progressive worsening of his symptoms. On arrival, he had decreased oxygen saturation, which improved with nasal cannula, and borderline elevation of blood troponin levels. D-dimer levels were elevated at >20 mg/ml prompting a CTA-PE protocol. It demonstrated bilateral lobar and segmental pulmonary emboli along with some of the typical lung parenchymal findings seen in COVID-19 (Figure 2).

Additionally, there was evidence of right heart strain, which was later confirmed by transthoracic echocardiography. He improved without requiring intubation.

Patient 3

**Figure 3:**
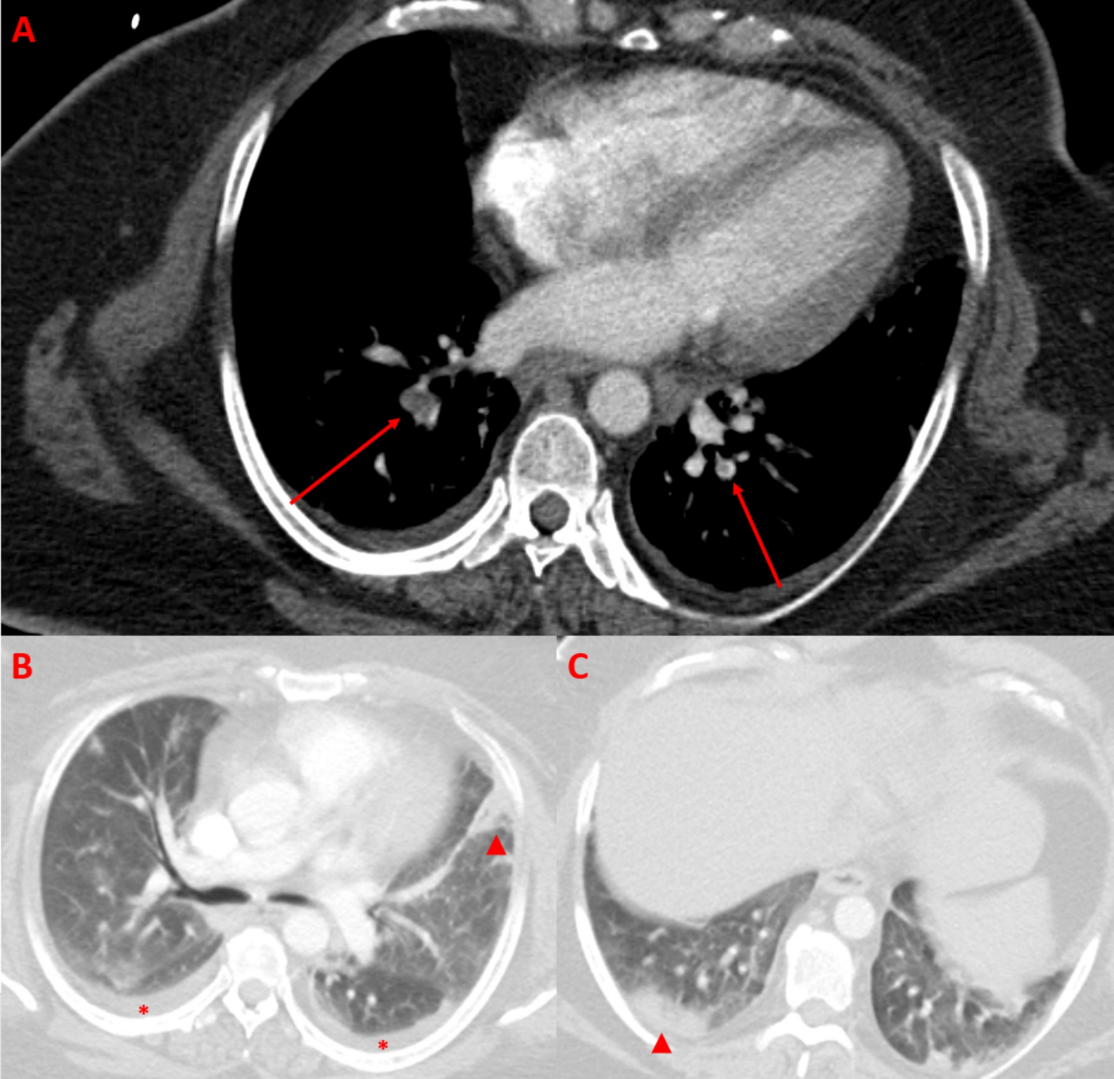
Axial CTA-PE views show filling defects in the bilateral lower lobe pulmonary arteries (arrows) representing pulmonary emboli (A). Lung windows show peripheral ground-glass and consolidative lung opacities (arrowheads). Also note bilateral pleural effusions (asterisk) (B, C). CTA-PE, CT angiogram pulmonary embolism

A 42-year-old female with obesity, obstructive sleep apnea, diabetes mellitus, and thalassemia trait presented to the emergency room and was admitted after a positive outpatient SARS-CoV-2 test and progressive shortness of breath. She had Staphylococcus epidermidis bacteremia and was treated with broad-spectrum antibiotics, azithromycin, and hydroxychloroquine. On hospital day 2, she was intubated for progressive hypoxic respiratory failure and slowly improved on ventilatory support. Tocilizumab treatment was added. Her D-dimer was modestly elevated to 1.56 mg/ml on admission to the ICU but had increased throughout the week up to 8.8 mg/ml, prompting heparin treatment for possible PE. After extubation, a CTA-PE protocol confirmed bilateral lower lobe segmental pulmonary emboli along with peripheral ground-glass opacities (Figure 3).

## Discussion

Thromboembolic disease is a significant cause of morbidity and mortality [2]. There have now been multiple reports of PE associated with COVID-19 [1,3,4]. Other thromboembolic complications have also been detailed including myocardial infarction, ischemic stroke, and DVT [4]. For example, in an analysis of 88 patients with COVID-19, Wang et al. found that 19 (22%) had developed DVT [5].

It has recently been suggested that empiric anticoagulation may improve prognosis in COVID-19-associated coagulopathy (CAC) [6]. Coagulopathy in COVID-19 may be multifactorial in etiology, related to sepsis, and/or unique to SARS-CoV-2 [7]. There is some evidence that features of CAC differ from non-SARS-CoV-2 causes of sepsis with disseminated intravascular coagulopathy (DIC). One study found that thrombocytopenia is milder in CAC compared with severe pneumonia from other causes [8]. Additionally, D-dimer elevation is often higher in CAC [9]. Patients 1 and 2 had peak levels of D-dimer >20 mg/ml, while patient three had a peak of 8.8 mg/ml. One recent report describes a significantly higher mortality among patients with levels of D-dimer ≥2.0 mg/ml on admission, although it is unclear if this higher mortality is directly caused by thromboembolic complications [10]. In contrast, patient 2 had a very elevated D-dimer but did not become critically ill and improved without requiring intubation or mechanical ventilation. None of our patients had a known clinical history of coagulopathy, risk factors, or history of thromboembolic disease prior to infection with SARS-CoV-2.

PE commonly presents with shortness of breath, tachycardia, cough, and/or pleuritic chest pain. These are also cardinal symptoms in COVID-19 pneumonia, and frequent in patients with severe bacterial pneumonia associated with shock. This symptom overlap may decrease the clinical suspicion for PE. However, when unexplained obstructive shock and new hemoptysis are present, PE is more likely to be considered [11,12]. Baseline screening and subsequent monitoring of coagulation tests, such as D-dimer, may also help to identify the development of coagulopathy and PE, although this requires further validation [13].

All three patients had pulmonary emboli and peripheral ground-glass opacities on imaging with the CTA-PE protocol. Ground-glass opacities are frequent findings in COVID-19 pneumonia, but can also directly result from PE [14]. If the diagnosis of COVID-19 has not been established, these findings may solely be attributed to PE. In contrast, if a non-contrast CT was performed, PE may not have been considered in patients with ground-glass opacities and known COVID-19. This is especially true early in the disease process when lung findings may be less pronounced. Other typical pulmonary findings described in COVID-19 pneumonia, such as interlobular septal thickening and crazy-paving pattern, were absent in these cases. Pleural effusions, such as those seen in patients 1 and 3, are also uncommon in COVID-19 but can be seen in progressive disease and are very commonly present in conjunction with PE [15].

## Conclusions

Thromboembolic disease with PE appears to be associated with severe COVID-19. Prompt recognition is important, but the diagnosis can be overlooked given the clinical overlap of PE and COVID-19 with sepsis. Evaluation with a CTA-PE protocol may be beneficial for selected patients presenting with clinical deterioration to exclude thromboembolic disease and PE.
